# Understanding the Pathological Basis of Neurological Diseases Through Diagnostic Platforms Based on Innovations in Biomedical Engineering: New Concepts and Theranostics Perspectives

**DOI:** 10.3390/medicines5010022

**Published:** 2018-02-25

**Authors:** Laura Ganau, Lara Prisco, Gianfranco K.I. Ligarotti, Rossano Ambu, Mario Ganau

**Affiliations:** 1 School of Medicine, University of Cagliari, 09124 Cagliari, Italy; lolly26it@yahoo.it (L.G.); amburo@unica.it (R.A.); 2 John Radcliffe Hospital, Oxford University, Oxford OX3 9DU, UK; lara.prisco@ndcn.ox.ac.uk; 3 Fondazione IRCCS Istituto Neurologico “Carlo Besta”, 20133 Milano, Italy; gianfrancokiligarotti@gmail.com; 4 Department of Neurosurgery, Toronto Western Hospital, University of Toronto, Toronto, ON M5T 2S8, Canada

**Keywords:** biomarkers, proteomics, nanotechnology, precision medicine, neurosurgery

## Abstract

The pace of advancement of genomics and proteomics together with the recent understanding of the molecular basis behind rare diseases could lead in the near future to significant advances in the diagnosing and treating of many pathological conditions. Innovative diagnostic platforms based on biomedical engineering (microdialysis and proteomics, biochip analysis, non-invasive impedance spectroscopy, etc.) are introduced at a rapid speed in clinical practice: this article primarily aims to highlight how such platforms will advance our understanding of the pathological basis of neurological diseases. An overview of the clinical challenges and regulatory hurdles facing the introduction of such platforms in clinical practice, as well as their potential impact on patient management, will complement the discussion on foreseeable theranostic perspectives. Indeed, the techniques outlined in this article are revolutionizing how we (1) identify biomarkers that better define the diagnostic criteria of any given disease, (2) develop research models, and (3) exploit the externalities coming from innovative pharmacological protocols (i.e., those based on monoclonal antibodies, nanodrugs, etc.) meant to tackle the molecular cascade so far identified.

## 1. Introduction

Over the last two decades, the continuous research efforts in the area of neurosciences have progressively expanded our understanding of many pathologies affecting the central and peripheral nervous systems (CNS and PNS, respectively). This trend is coupled with exponential advances in other basic sciences, particularly nanotechnology and biomedical engineering [1]. The result of these endeavors in the basic sciences and their confluence into new translational projects, theorized and implemented by enlightened multidisciplinary research teams, is now opening up unexpected horizons in clinical practice, heading toward diagnosis and treatment at the nanoscale of many pathological conditions [1,2]. In some cases, this paradigmatic change has already occurred; in the majority of cases, this paradigm shift will become increasingly evident in the next few years.

Innovative diagnostic platforms based on biomedical engineering (microdialysis and proteomics, biochip analysis, non-invasive impedance spectroscopy, etc.) are introduced at a rapid speed in clinical practice: this article primarily aims to highlight how such platforms will advance our understanding of the pathological basis of neurological diseases; an overview of the clinical challenges and regulatory hurdles facing their introduction in clinical practice, as well as their potential impact on patient management, will complement the discussion on foreseeable theranostics perspectives.

## 2. Microdialysis and Proteomics

A smart technique used for the sampling and identification of small-molecular-weight substances in the CNS interstitial space is represented by microdialysis. Due to its versatility, the microdialysis technique is employed in a number of areas in biomedical research:for instance, in neuroscience, this technique has helped to advancing our knowledge of many synaptic pathways given the accurate collection and quantification of neurotransmitters, peptides, and hormones under physiological and pathological conditions. Moreover, in clinical practice, the technique moved forward the monitoring of brain injuries, intracranial hemorrhages, and tumors. However, one of the limitations underlying the microdialysis process consists in the difficulty of effectively estimating the extracellular concentration of the protein of interest from dialysis samples (a concept known as relative recovery) in most in vivo studies. In fact, the concentration of a substance obtained directly from the microdialysis technique does not accurately describe the concentration of the substance on-site [3]. In order to relate the results collected from microdialysis to the actual in vivo conditions, or to relate the in vivo relative recovery to data interpretation, calibration methods are required. As such, several methodologies to optimize the analysis of the smallest volumes (to a few microliters) obtained during sampling have long been investigated, and these efforts have led to the advent of new proteomic techniques.

Proteomics in its broadest mandate investigates the presence of proteins in intracellular and extracellular space, and their functions. Following transcription of the information encoded into our DNA, the expression of peculiar proteins regulates the cellular structure and function, including migration, interactions, and longevity. The proteome is highly variable not only from physiological to pathological conditions but also from person to person, and from cell to cell. It is in fact vastly more complex than the corresponding genome, and it is fair to say that alterations in protein functions eventually regulate the onset or progression of any disease. Therefore, an understanding of protein networks through a systems biology approach of proteomics is necessary to understand normal and abnormal cellular function with the goal of performing rational therapeutic interventions [4,5].

As an evolving technology, proteomics has benefited from developments in mass spectroscopy, atomic force microscopy, and other high throughput analytical tools in conjunction with bioinformatics analysis [4]. It is noteworthy that the promise of individualized molecular medicine seems particularly relevant in two fields: (a) neuro-oncology, where similarly classified tumors can show quite different clinical behavior and aggressiveness, and (b) neuro-traumatology, where many patients appearing and behaving very similarly at baseline can present a diverse range of clinical outcomes due to evolving functional deficits [6,7]. In both cases, identifying molecular targets for the early diagnosis of pathological conditions can provide useful prognostic information and meaningfully enhance their therapeutic management.

## 3. Biochip Analysis

**Figure 1:**
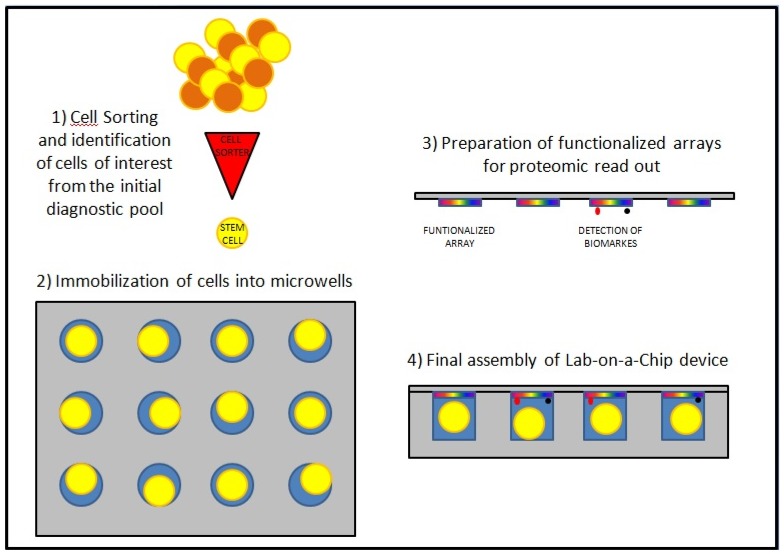
Schematic assembly of a lab-on-a-chip device. The initial cell sorting allows for the identification of relevant cells, such as cancer stem cells (CSCs), thanks to the recognition of superficial antigens; following immobilization of those cells into microwells, the step of biomarkers identification is obtained through functionalized arrays positioned on top of each microwells.

From the perspective of clinical diagnostics, biochip analysis on a multicellular level has been a well accepted approach in several fields: for example, in microbiology, oral bacterial infections can now be detected or followed up with expression chips used as point-of-care diagnostics. Nonetheless, going forward, the greatest expectations are related to a better analysis of key single cells. The added value of single cell analysis becomes clear when it is considered that large amounts of cells sampled by microdyalisis or multicell proteomic techniques are usually a mixture of different cell types and sometimes a mixture of the same cells that show either healthy or pathological conditions. As a result, the acquisition of statistically significant results has been extremely difficult for a long time; while a better understanding of disease etiology, carcinogenesis, and progression has been gained only with the ability to identify (thanks to cell sorting methodologies) and study one specific type of cells, their function as building blocks in the tissues and organisms, and thus their role in cell–cell interaction, migration, differentiation, etc. [8,9]. This became particularly relevant when scientists and clinicians started to focus their attention on cancer stem cells (CSCs). It is noteworthy that several techniques for the study of single cells, including cloning rings, laser microdissection, and live-cell catapulting are now available for the isolation of single adherent cells; while magnetic sorting, column chromatography, and various microfluidic approaches are commonly used for non-adherent cells [5,10]. Whatever the approach, the final aim is to incorporate the technique of choice into a lab-on-a-chip device (Figure 1) meant for multiplexing analysis of different biomarkers from the proteome of previously selected cells of interest [6].

In recent years, the best example of this breakthrough approach was probably the commercialization of kits for robust and reproducible detection of circulating cancer cells (CTCs) from a simple blood test. For simplicity, these methodologies can be divided into nucleic-acid-based (relying on the detection of specific DNA or RNA sequences differentially expressed by tumor cells) and cytometric (based on immunomagnetic separation, identification, and enumeration of CTCs through fluorescence microscopy or immunohistochemistry) approaches. The study of CSCs and CTCs offered a new opportunity to learn more about the biology of primary tumors and metastases, and in the near future could serve to reveal their response/resistance to various chemotherapy protocols [11].

Over the years, further improvements came whenever those methodologies increased their sensitivity, leading to the full expansion of their role. Emerging solutions provided by platforms based on biomedical engineering amplified the range of different molecules studied beyond DNA and proteins, such as messenger RNA (mRNA), microRNA (miRNA), exosomes, and microvescicles, thus playing a key role in the discovery and characterization of biomarkers and biosignatures for early disease detection, subclassification, and the predictive capability of current proteomics modalities [12].

Moreover, by miniaturizing and functionalizing the surface of biochips via nanotechnological methods, scientists have been able to design even smaller probes, which has fundamentally resulted in the study of the cells’ behavior under pathologic conditions. For example, the cellular crosstalk by expression of exosomes or secreting microvescicles, which eventually make their way into the circulation, interact with each other, and directly affect pivotal metabolic pathways, has been known for a while. Only recently, however, their role in cancer and trauma has been at the center of investigation through mass spectrometry techniques used in combination with mathematical algorithms typical of systems biology [13,14]. Thus, we now have substantial evidence suggesting that low-molecular-weight circulatory proteomes also contain information that can be used to detect diseases in their preclinical state [15,16,17].

Supported by integrated nanoanalytical models, the identification of pivotal features of disease is expected to grow not only for the degenerative or infective pathologies mentioned above but also for rare ones (i.e., orphan diseases), overcoming the barrier previously represented by the low incidence and undetectable peculiar biomarkers/biosignatures in conventional diagnostics, which has kept such disease features hidden for a long time.

## 4. Optical Imaging and Other Non-Invasive, Real-Time Diagnostics Strategies

Beside molecular diagnostics, cellular imaging represented a major breakthrough in the neurosciences. Optical imaging exploits optically labeled targeting agents and leverages on the unique light emission of tissues in physiological or pathological states [1,18,19,20,21]. Since almost two decades, neurosurgeons started to rely on various dyes such as 5-aminolevulinic acid, commonly known as 5-ALA, in malignant glioma surgery: during the surgical procedure, this dye is administered endovenously to optimize complete resection with intralesional margins [18,19,20]. In fact, highly vascularized tumors appear fluorescent under violet-blue excitation light (600 nm), and this allows for the identification of tumor infiltration in the surrounding parenchyma that would have been unrecognizable under white light [20,21]. Compared to visible light, near-infrared (700–900 nm) imaging of fluorescent probes exhibitssignificantly superior tissue penetration (5–10mm) with little interference from fluorescence emanating from endogenous fluorophores. Minimally invasive surgical approaches certainly represent the ideal setting for use of near-infrared (NIR) imaging in oncologic surgery for the following reasons: a low ambient light environment and the incorporation of fluorescence imaging systems into existing operative microscopes [22].

Furthermore, novel strategies to measure changes in the electrical impedance spectrum within the brain using shielded scalp electrodes opened new doors in the understanding and management of various neurological pathologies, including stroke and epilepsy. This non-invasive approach to monitoring brain functions is based on the principle that acute hemorrhagic stroke or epileptic fits may produce detectable changes in the impedance spectrum measured on the subject’s scalp due to parenchymal local increases of blood volume [23,24]. Of note, this innovation can soon translate into a remarkable improvement in neuromonitoring, so that the electrical properties of the brain of patients with traumatic brain injuries admitted to NeuroIntensive Care Units would be continuously and non-invasively assessed by spectral electrical impedance estimation [7].

## 5. Theranostics

**Figure 2:**
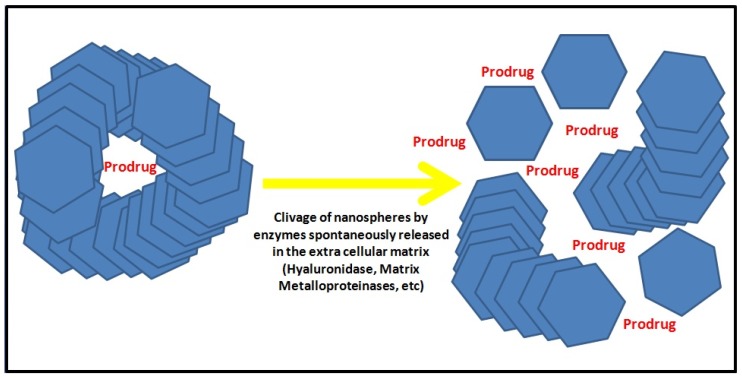
Protease-activated drug delivery. Following identification of their targets, the release of a prodrug encapsulated in theranostic agents, based for instance on nanospheres of hyaluronic acid or quantum dot gelatin nanoparticles, is obtained through cleavage of their scaffold by enzymes highly expressed in tumors (such as hyaluronidase or matrix metalloproteinase 2).

Interestingly, the possibility of directly probing cellular properties, controlling and intensifying in real time their physical and chemical processes during biological events, has yielded an interest in treating pathological conditions. In many neurological diseases, ranging from brain tumors to CNS-HIV infections, miniaturization to the nanometer scale now allows for improved biodistribution and target site accumulation of systemically administered drugs [25,26]. Such miniaturization now constitutes a giant leap forward, since scientists and clinicians have started collaborating to transform conventional approaches used for decades in clinical imaging and drug delivery (Figure 2).

Different types of nanodrugs and enhanced contrast agents have been evaluated over the years, including, for instance, liposomes, polymers, micelles, and antibodies, and a significant amount of evidence has been obtained showing that these submicrometer-sized carriers are able to improve the efficacy of therapeutic interventions [27]. In neuro-oncology, one of the key benefits of optimizing contrast agents with nanoparticle-based materials (such as quantum dots, mesoporous silica nanoparticles, and carbon-based nanoparticles) is the ability to overcome the blood–brain barrier and directly visualize cancer cells; this has great repercussion, for instance, on the detection of recurrent tumors, whose infiltration within the surrounding brain is not detectable with conventional imaging techniques [28]. Furthermore, such an approach has several externalities in the management of non-tumoral conditions, such as pseudoprogression and radionecrosis, and in general postoperative imaging changes [29]. The greatest advantage of combining diagnostic and therapeutic agents within a single formulation is certainly the increased biocompatibility and tailored delivery: beyond the improved pharmacokinetics, this strategy is in fact extremely promising in terms of safety due to the reduced off-target accumulation in healthy organs [30]. By coupling therapeutics within novel diagnostic agents, meant to yield a higher photoluminescence and photostability, microscopic imaging is expected not only to offer better direct visualization of cells and molecules but also to soon provide the means to target specific cells and finely control the delivery of chemotherapies, radioenhancers, and radiosensitizers [31,32].

Optogenetics, a neuromodulation method that exploits the use of visible light to control living neurons that have been genetically modified to express light-sensitive ion channels, deserves mentioning [33,34]. The performance of optoelectronic microprobes has been enhanced, in both acute and chronic implantations, by adopting nanomaterials whose properties (i.e., spatial resolution, interactions with the target tissue, etc.) allow for optimization of light delivery to the tissue, either by waveguides or by integrated light sources at the sites of intervention [35,36,37]. Another example of the advances in optogenetics through nanotechnology and biomedical engineering is the use of upconversion nanoparticles (UCNP), which are able to absorb tissue-penetrating NIR light and emit wavelength-specific visible light; use of such light has been advocated as being able to overcome the initial limitationof using visible light, which obviously cannot penetrate deep inside the brain tissue [38]. In an experimental setting, transcranial NIR UCNP-mediated optogenetics evoked a dopamine release from genetically tagged neurons, silenced seizures by inhibition of hippocampal excitatory cells, and triggered memory recall [38].

The techniques described in this section share a common goal: reducing the risk of side effects or unwanted toxicity and enabling less invasive cell manipulation, with the potential for remote therapy. Fostering this kind of approach will eventually lead to the clinical use of the circulating nanorobots described below.

## 6. Outlook on Neuro-Nanorobots for Diagnostic Purposes

The trend outlined above perfectly justifies the introduction of neuro-nanorobots in the field of neurology and neurosurgery, as they promise to enrich our diagnostic and therapeutic armamentarium. Achievements in miniaturizing chip technology, along with progress in optics and micro-mechanics, have allowed for the development of micro- and nanosized robots for use in numerous biomedical applications, such as monitoring diagnoses and the repair and treatment of human biological systems [2,39]. For instance, nanoelectronic chemical sensors have been embedded into microrobotic prototypes programmed for the proteomic detection of different intravascular levels of NOS: by detecting abnormal values, it would in fact be possible to interpret the early patterns of development and growth of intracranial aneurysms. More importantly, such robots could alert the treating physician through radiofrequency wireless communication and eventually replace the need for serial follow-up with angioCT scans [40]. Similarly, other teams are now working on swarms of propelled nanorobots able to recognize cancer cells within the CNS and to forward such information to the clinicians through acoustic signals in a distributed and decentralized fashion [41]. Given the striking pace of advancement of these technologies, the translation of such prototypes from a laboratory setting to clinical wards will only require a few years, provided that initial satisfactory results are confirmed in randomized controlled trials.

## 7. Challenges and Hurdles

Biomedical engineering has already demonstrated its potential to be a game-changer in clinical practice, with the promise to optimize the omni-comprehensive management of many pathological conditions. In this perspective article, we have pinpointed why these innovations will push forward the boundaries of our knowledge of the pathological basis of diseases with remarkable improvements in our diagnostics and therapeutic capabilities. However, the challenges facing these methodologies are multifold: they include safety and toxicology concerns, manufactory costs, and regulatory issues.

The conception, design, and testing of more effective diagnostic techniques is the core of innovation and benchmarking: to increase the potential for clinical translation, researchers are exploring new materials and creating relevant animal disease models. Such remarkable research efforts are critical for attaining specific and sensitive information that allows subsequent comparisons between different diagnostic strategies. As per innovative contrast agents and theranostics, some of the specific challenges are associated with safety concerns requiring ad hoc toxicology studies, or the need for validation by the international community through well designed randomized controlled trials [42,43].

Hence, while preclinical data supports the use of many of these methodologies, the translational hurdles posed by consistent research anddevelopment costs have limited their translation in clinical practice: in fact, the industry is often reluctant to support clinical translation in light of the rising costs of adhering to guidelines for investigational and therapeutic molecules. Of note, revenues associated with diagnostic agents are just a fraction of those coming from therapeutic agents despite similar initial investments for approval by national and international regulatory bodies, such as the United States Food and Drug Administration (FDA), the European Medicines Agency (EMEA), the Biologics and Genetic Therapies Directorate (BGTD) of Health Canada, the China Food and Drug Administration (CFDA), and the Australian Therapeutic Goods Administration (TGA).

## 8. Conclusions

In summary, this article demonstrates how innovative diagnostic platforms based on biomedical engineering are being introduced at a rapid speed in clinical practice; we will probably witness in the coming years a revolution in our ability to (1) identify biomarkers that better define the diagnostic criteria of any given disease, (2) develop research models, and (3) exploit the externalities coming from innovative pharmacological protocols (i.e., those based on monoclonal antibodies, nanodrugs, etc.) meant to tackle the molecular cascade so far identified.

As such, what is not considered treatable today may be deemed so, if not curable, in the span of few generations. The two systematic reviews prepared for this special issue, *New Innovations in Biomedical Engineering*, will further expand on why this is the case in neuro-oncology and neuro-traumatology [44,45].
